# The Diagnostic Accuracy of Hyperbilirubinemia in Predicting Appendicitis and Appendiceal Perforation

**DOI:** 10.7759/cureus.48203

**Published:** 2023-11-03

**Authors:** Syed Yousaf Khalid, Aiman Elamin

**Affiliations:** 1 Urology, Letterkenny University Hospital, Letterkenny, IRL; 2 General Surgery, Letterkenny University Hospital, Letterkenny, IRL

**Keywords:** white blood cell count, c-reactive protein, hyperbilirubinemia, appendectomy, appendicitis

## Abstract

Background

Every diagnostic tool that may assist in the identification of appendicitis is of great importance to emergency general surgeons. While recent research has indicated that hyperbilirubinemia can serve as a valuable predictor of appendiceal perforation, these studies have not specifically examined the role of bilirubin as an indicator for acute appendicitis. This study aimed to assess the role of hyperbilirubinemia as a diagnostic factor in detecting appendicitis and appendiceal perforation.

Methodology

This single-center retrospective study involved 333 patients with acute appendicitis who underwent an emergency appendectomy at a model three hospital between January 2021 and December 2022. Statistical analysis was performed using STATA/SE 18.0 for Windows (StataCorp., College Station, TX, USA) to compare bilirubin levels, white blood cell count (WCC), and C-reactive protein (CRP) among normal appendices, non-perforated appendicitis, and perforated appendicitis.

Results

Among 333 patients, 60.66% were male, and 39.34% were female, with a male-to-female ratio of 1.54:1. The average hospital stay was 3.27 ± 3.02 days. Hyperbilirubinemia was observed in 25.53% (85 individuals). Among the 51 cases of perforated appendicitis, 70.59% had elevated bilirubin levels of above 20 μmol/L. Significantly more patients with appendiceal perforation had hyperbilirubinemia than non-perforated appendicitis (70.59% vs. 19.03%, p < 0.001).

Bilirubin had higher specificity (94.29%) for detecting non-perforated appendicitis than normal appendices (odds ratio = 3.88), while WCC and CRP showed higher sensitivities. WCC had a sensitivity of 73.28% and a specificity of 42.86%, and CRP had a sensitivity of 76.53% and a specificity of 60.00%.

When comparing perforated appendicitis with non-perforated appendicitis, bilirubin showed a specificity of 80.97% and an odds ratio of 10.21. The likelihood of a patient with hyperbilirubinemia having perforated appendicitis was more than 10 times higher than those without appendiceal perforation, suggesting it to be a potential indicator for appendiceal perforation. WCC showed a specificity of 26.72% and an odds ratio of 4.28, while CRP had a specificity of 23.48% and an odds ratio of 4.91.

Conclusions

The significant association between bilirubin levels and appendicitis highlights its potential as a valuable marker for predicting appendicitis and appendiceal perforation. The simplicity, cost-effectiveness, and diagnostic value of bilirubin assessment support its routine use in suspected cases of acute appendicitis.

## Introduction

The vermiform appendix is a tiny blind-ended pouch connected to the large intestine. Vermiform denotes “worm-like,” which defines its slender, tubular shape [1]. The importance of the vermiform appendix in the field of surgery stems mainly from its tendency to undergo inflammation, resulting in a condition called acute appendicitis. Acute appendicitis is commonly caused by blockage of the lumen of the appendix by fecalith, normal stool, infectious agents, or lymphoid hyperplasia and can be worsened by polymicrobial infection. Perforation, peritonitis, and intra-abdominal abscesses are among the complications [2,3].

Acute appendicitis is a common condition, with more than 40,000 annual hospital admissions in England [4]. The lifetime risk is higher in men (8.6%) than in women (6.7%), with the highest occurrence (approximately 40%) in the second decade of life (10-19 years) and 70% of cases occurring in individuals under 30 years of age [5]. Non-perforated appendicitis has a low mortality rate (0.1-0.5%), whereas perforated appendicitis has a significantly higher mortality rate (3% in general to 15% in elderly patients) [6]. Perforation rates range from 16% to 40%, with higher rates in younger age groups (40-57%) and in those aged >50 years (55-70%) [7,8].

Diagnosing acute appendicitis is challenging and relies on various factors such as patient history, physical examination, laboratory tests, and imaging. Key symptoms include abdominal pain near the navel, loss of appetite, nausea, pain shifting to the lower right abdomen, and a mild temperature increase [9]. However, elderly patients may exhibit atypical or diminished symptoms, whereas symptoms in children are often non-specific because of communication limitations [10]. This complexity can cause diagnostic confusion and delays. Appendicitis may mimic various medical conditions (e.g., diverticulitis, mesenteric adenitis, Crohn’s disease, endometriosis, ectopic pregnancy, omental torsion, pelvic inflammatory diseases, ruptured ovarian cysts, and urinary tract infections), leading to diagnostic challenges. The global diagnostic error rate difference between men and women is 12-23% and 24-42%, respectively [11].

Delayed surgical intervention in appendicitis patients, especially those without perforation, can lead to severe complications such as gangrene and perforation. Appendiceal perforation may result in additional issues, such as abscess formation, peritonitis, fecal fistulas, intestinal obstruction, portal pyemia, sepsis, and potential infertility in women [4]. Conversely, when faced with diagnostic challenges and atypical presentations, performing appendectomy solely based on clinical suspicion may lead to up to 20% of unnecessary surgeries [12]. Unwarranted appendectomy carries the risk of wound sepsis, intestinal obstruction due to adhesions, and incisional hernia development.

Although blood tests can assist in the diagnosis of acute appendicitis, clinical expertise remains vital. No definitive or specific markers of acute appendicitis have been identified. An elevated white cell count (WCC) is not exclusive to appendicitis. C-reactive protein (CRP) is frequently used in suspected appendicitis, but its specificity varies across studies [13]. Several scoring systems, such as the Alvarado and Modified Alvarado scores, aid in diagnosis. Radiological modalities such as CT or MRI do not effectively distinguish between complicated and uncomplicated appendicitis [14]. Additional diagnostic tools are required to complement clinical examinations and radiology to enhance the chances of early detection.

This study aimed to investigate the association between hyperbilirubinemia and appendicitis severity before surgical intervention. We analyzed the diagnostic value of bilirubin in distinguishing appendicitis from normal appendices by using data from patients who underwent emergency appendectomy. Additionally, we explored the link between hyperbilirubinemia and perforated appendicitis and compared the specificity of bilirubin with WCC and CRP levels for diagnosing both non-perforated and perforated appendicitis.

## Materials and methods

We conducted a retrospective review of all appendectomies performed between January 2021 and December 2022 in the general surgery department of a model III hospital in Ireland. The Hospital Inpatient Enquiry department provided the list of patients who underwent an urgent appendectomy by any method during the study period. Data, including demographic information, blood investigations, and histopathological reports, were extracted from the computerized hospital information system. Patients were considered for inclusion in the study if they had an urgent appendectomy performed with postoperative histopathological evaluation of the appendix specimen and had their WCC, CRP level, and serum bilirubin level checked on admission. Exclusion criteria were applied to eliminate subjects with incomplete medical records; missing WCC, CRP, or serum bilirubin levels on admission; or had specific medical conditions that could confound the results such as a history of chronic liver disease with hyperbilirubinemia, chronic alcoholism, hemolytic disease, biliary disease, acute hepatitis, gastrointestinal malignancy, and hepatotoxic drug use. Consecutive sampling was employed and a sample size of 389 patients was determined based on the data available during the study period. A total of 333 patients were included according to the inclusion criteria. Statistical analyses were performed using STATA/SE 18.0 for Windows (StataCorp., College Station, TX, USA). Descriptive statistics were used to summarize continuous variables and were expressed as mean ± standard deviation. Categorical variables were expressed as absolute numbers and percentages. The chi-square test was used to assess the relationships between categorical variables. Diagnostic accuracy measures, including sensitivity, specificity, positive predictive value (PPV), and negative predictive value (NPV), were calculated with the corresponding 95% confidence intervals. Statistical significance was set at a p-value <0.05.

Patients were categorized based on the histopathological results of the appendix samples into the following five groups: normal appendices, acute focal appendicitis, acute suppurative appendicitis, gangrenous appendicitis, and perforated appendicitis.

Three broad patient groups were compared: normal appendices were compared with non-perforated appendicitis (including acute focal, suppurative, and gangrenous appendicitis), and appendiceal perforation was compared with non-perforated appendicitis. The mean bilirubin levels, WCC, and CRP levels in each group were compared. The comparison was also made regarding elevated levels of these potential markers. The sensitivity, specificity, PPV, NPV, and odds ratio of hyperbilirubinemia, increased CRP level, and WCC for non-perforated and perforated appendicitis were calculated.

A bilirubin level over 20 μmol/L was defined as hyperbilirubinemia, a raised WCC greater than 11 x 10^9^/L, and a raised CRP level greater than 10 mg/L.

## Results

A total of 389 patients underwent an appendectomy between January 2021 and December 2022. From this pool, 333 patients who met the predefined inclusion criteria were included in the analysis. The average length of hospital stay for these patients was 3.27 ± 3.02 days. The negative appendectomy rate was 10.51% in our study.

Gender-specific characteristics

Among the 333 patients included in the study, 60.66% were male and 39.34% patients were female, resulting in a male-to-female ratio of 1.54:1. The overall occurrence of perforated appendicitis was 15.32%, with 33 cases occurring in males and 18 in females out of a total of 51 cases. On the other hand, most patients (74.17%) had non-perforated appendicitis, with 156 cases in males and 91 cases in females out of the total 247 cases. Notably, among the 35 patients with a normal appendix, according to the histopathology report, the percentage of female patients (62.86%) was higher than that of the male patients (37.14%).

Age-specific characteristics

The age of the patients ranged from 3.37 to 87.03 years, with an overall average age of 31.39 ± 20.03 years. The mean age for males was 30.99 ± 20.12 years, and for females, it was 32.03 ± 19.96 years.

**Figure 1:**
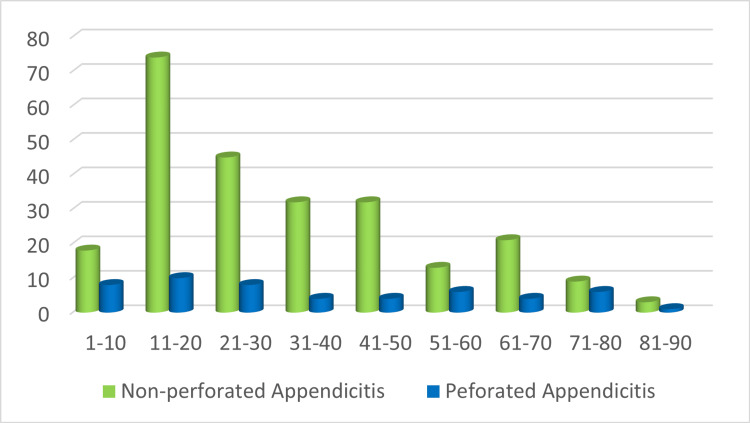
Frequency distribution of non-perforated and perforated appendicitis among the various age groups.

Figure 1 presents a comprehensive overview of the frequency distribution of non-perforated and perforated appendicitis among the various age groups. In the case of non-perforated appendicitis, the 11-20-year age group exhibited the highest frequency, accounting for 74 cases, or 29.96% of the total non-perforated appendicitis cases. The 21-30-year age group had the second highest frequency, comprising 45 cases, or 18.22% of the total.

Likewise, the 11-20-year age group demonstrated the highest frequency, with 10 cases of perforated appendicitis, accounting for 19.61% of the total perforated appendicitis cases. Notably, the 1-10 and 21-30 age groups shared the second highest frequency, each accounting for eight cases, or 15.69% of the total.

Categorization of data and comparative analysis

Table 1 summarizes the distribution of different types of appendices and the occurrence of hyperbilirubinemia within each group. The table includes the number of patients and their respective percentages for each category. The data indicates that as the severity of appendicitis increased, the percentage of patients with hyperbilirubinemia also increased.

**Table 1:** Distribution of different types of appendices and the occurrence of hyperbilirubinemia within each group.

Types of appendices	Number of patients (% of total)	Number of patients with hyperbilirubinemia (% of group)
Normal appendices	35 (10.51%)	2 (5.71%)
Acute focal appendicitis	78 (23.42%)	5 (6.41%)
Acute suppurative appendicitis	130 (39.04%)	29 (22.30%)
Acute gangrenous appendicitis	39 (11.71%)	13 (33.33%)
Perforated appendicitis	51 (15.32%)	36 (70.59%)
Total	333	85

To further analyze the data, we categorized them into three groups: normal appendices, non-perforated appendicitis, and perforated appendicitis. We then conducted a comparative analysis between non-perforated appendicitis and normal appendices and between perforated and non-perforated appendicitis cases.

**Figure 2:**
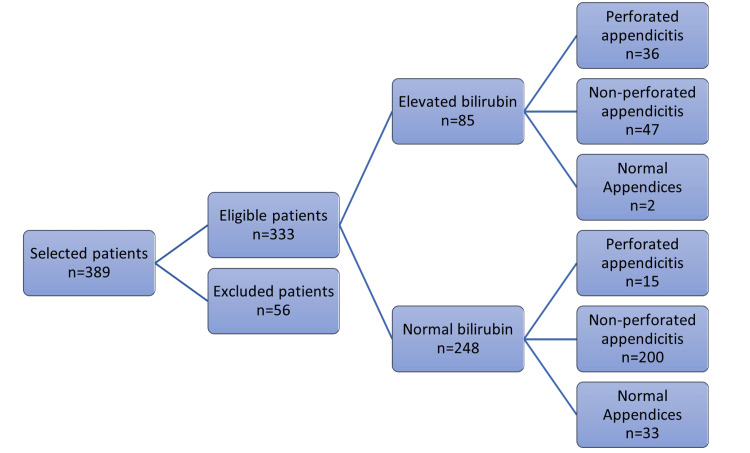
Classification of patients based on bilirubin levels and appendicitis status.

We explored the relationship between bilirubin levels and different categories of appendicitis (Figure 2). Of the 333 patients, 85 (25.53%) exhibited hyperbilirubinemia, with serum total bilirubin levels exceeding 20 μmol/L. Among the 51 patients diagnosed with perforated appendicitis, 36 (70.59%) had elevated total bilirubin levels >20 μmol/L. Conversely, the remaining 15 (29.41%) participants had normal levels (<20 μmol/L). Significantly more patients with appendiceal perforation had hyperbilirubinemia than those with non-perforated appendicitis (70.59% vs. 19.03%, p < 0.001).

Statistical analysis revealed that bilirubin demonstrated a higher specificity (94.29%) in detecting non-perforated appendicitis than in detecting a normal appendix. The odds ratio for bilirubin in relation to non-perforated appendicitis was 3.88. Conversely, WCC and CRP showed higher sensitivity in detecting non-perforated appendicitis. The sensitivity of WCC for non-perforated appendicitis was 73.28%, whereas that for CRP was 76.53%. The specificities of WCC for non-perforated appendicitis was 42.86% and that for CRP was 60.00% (Table 2).

**Table 2:** Bilirubin vs. WBC vs. CRP for non-perforated appendicitis vs. normal appendices. 1*: normal appendices; 2*: non-perforated appendicitis; WCC: white cell count; CRP: C-reactive protein; PPV: positive predictive value; NPV: negative predictive value

Non-perforated appendicitis vs. normal appendices						
Bilirubin		(2)*	(1)*	Specificity	94.29%	Odds ratio: 3.88
	Positive	47	2	Sensitivity	19.03%	
	Negative	200	33	PPV	95.92%	
				NPV	14.16%	
WCC		(2)*	(1)*	Specificity	42.86%	Odds ratio: 2.06
	Positive	181	20	Sensitivity	73.28%	
	Negative	66	15	PPV	90.04%	
				NPV	18.52%	
CRP		(2)*	(1)*	Specificity	60.00%	Odds ratio: 4.88
	Positive	189	14	Sensitivity	76.53%	
	Negative	58	21	PPV	93.10%	
				NPV	26.58%	

When comparing perforated appendicitis with non-perforated appendicitis, bilirubin level exhibited a specificity of 80.97% and an odds ratio of 10.21. This indicates that the likelihood of a patient with hyperbilirubinemia and perforated appendicitis is more than 10 times higher than that of a patient without appendiceal perforation. This finding suggests that hyperbilirubinemia can serve as a potential predictor of appendiceal perforation in patients with appendicitis. In contrast, WCC demonstrated a specificity of 26.72% and an odds ratio of 4.28, whereas CRP had a specificity of 23.48% and an odds ratio of 4.91 (Table 3).

**Table 3:** Bilirubin vs. WCC vs. CRP for perforated appendicitis vs. non-appendicitis. 2*: non-perforated appendicitis; 3*: perforated appendicitis; WCC: white cell count; CRP: C-reactive protein; PPV: positive predictive value; NPV: negative predictive value

Perforated vs. non-perforated appendicitis						
Bilirubin		3*	2*	Specificity	80.97%	Odds ratio: 10.21
	Positive	36	47	Sensitivity	70.59%	
	Negative	15	200	PPV	43.37%	
				NPV	93.02%	
WCC		3*	2*	Specificity	26.72%	Odds ratio: 4.28
	Positive	47	181	Sensitivity	92.16%	
	Negative	4	66	PPV	20.61%	
				NPV	94.29%	
CRP		3*	2*	Specificity	23.48%	Odds ratio: 4.91
	Positive	48	189	Sensitivity	94.11%	
	Negative	3	58	PPV	20.25%	
				NPV	95.08%	

Table 4 presents the results of the analysis of the three variables, i.e., bilirubin, WCC, and CRP. The table provides information on the means and standard deviations (SD) of these variables in the different groups.

**Table 4:** Biochemical values for each study group. P-values were calculated using the Kruskal-Wallis test. 1*: normal appendices; 2*; non-perforated appendicitis; 3*: perforated appendicitis; SD: standard deviation; WCC: white cell count; CRP: C-reactive protein

		1*	2*	3*	P-value
Bilirubin	Mean	8.91	13.35	25.41	<0.0001
	SD	4.99	8.78	19.27	
	Positive	2	46	35	
	Negative	33	201	16	
WCC	Mean	11.76	13.94	17.33	<0.0001
	SD	4.13	4.76	5.64	
	Positive	20	181	47	
	Negative	15	66	4	
CRP	Mean	26.27	57.09	125.26	<0.0001
	SD	47.01	65.60	95.19	
	Positive	14	189	48	
	Negative	21	58	3	

The mean bilirubin levels were significantly different across the different appendicitis categories. The mean bilirubin level for non-perforated appendicitis was 13.35 μmol/L, significantly higher than the mean bilirubin level for a normal appendices, which was 8.91 μmol/L. Furthermore, the mean bilirubin level for perforated appendicitis was higher at 25.41 μmol/L.

Regarding WCC, the mean value for a normal appendix was 11.76 x 10^9^/L. In comparison, non-perforated appendicitis had a mean value of 13.94 x 10^9^/L, and perforated appendicitis had a mean value of 17.33 x 10^9^/L.

For CRP levels, the mean value for a normal appendix was 26.27 mg/L. In contrast, non-perforated appendicitis had a mean value of 57.09 mg/L, and perforated appendicitis had a mean value of 125.26 mg/L. All these differences were statistically significant (p < 0.0001).

## Discussion

Appendicitis is a prevalent surgical emergency in modern medicine, with an annual occurrence rate of approximately 100 cases per 100,000 individuals [5]. Despite the increasing use of advanced imaging and non-invasive diagnostic techniques, such as ultrasound with graded compression, high-resolution helical CT, and laparoscopy, the rates of misdiagnosis of appendicitis (15%) and appendiceal rupture have remained unchanged [5]. These methods also have several noteworthy limitations, such as financial implications, exposure to radiation, operator dependence, allergy to contrasts, false-positive and false-negative diagnoses, and anesthesia exposure.

Recovery from emergency appendectomy for uncomplicated acute appendicitis is typically rapid but can be fatal if the condition is gangrenous or perforated. Perforated appendicitis carries the risk of severe complications, including bacterial peritonitis, urinary issues, small intestine blockage, and intra-abdominal abscess formation [15,16]. Rapid diagnosis is crucial to prevent such complications. However, no clinical or laboratory test can accurately predict complicated appendicitis during the initial stages. Therefore, there is a need to develop safe, affordable, accessible, and accurate diagnostic markers for early detection and management to reduce morbidity. Serum bilirubin estimation appears promising, fulfilling several of these requirements, and gaining popularity.

The significance of hyperbilirubinemia as a predictive factor of simple acute appendicitis is not widely acknowledged. Nevertheless, previous studies have found that hyperbilirubinemia is an indicator with a high specificity for perforated appendicitis. Our study showed that hyperbilirubinemia is a significant marker for simple acute appendicitis and not only for appendiceal perforation.

Patients with hyperbilirubinemia exhibited a notably higher likelihood of having uncomplicated acute appendicitis than those with normal bilirubin levels. Our study also found that hyperbilirubinemia can serve as a potential predictive factor of appendiceal perforation in patients with appendicitis. More patients with appendiceal perforation had hyperbilirubinemia than those with non-perforated appendicitis (70.59% vs. 19.03%, p < 0.001).

The prevalence rates of perforated appendicitis and non-perforated appendicitis were 15.32% and 74.17%, respectively. The high specificity value of 0.94 associated with hyperbilirubinemia in relation to non-perforated appendicitis serves as a significant indicator that should prompt clinicians to consider the likelihood of this diagnosis. Our secondary outcome confirmed the high specificity of bilirubin level (0.80) for appendiceal perforation. The odds ratio of 10.2 in our study was higher, indicating that the likelihood of a patient with hyperbilirubinemia having perforated appendicitis was more than 10 times greater than that for patients without appendiceal perforation.

Although bilirubin is more specific to appendicitis, WCC and CRP levels are more sensitive. This confirms the use of hyperbilirubinemia as a rule-in test and not as a rule-out test for appendicitis.

In a retrospective study by Sand et al. [17], elevated serum bilirubin levels showed a specificity of 86% for perforated appendicitis, surpassing the specificity of 35% for CRP. Our study also demonstrated a higher specificity of elevated bilirubin levels for perforated appendicitis (80.97%) than CRP (26.72%). In differentiating non-perforated appendicitis from normal appendices, serum bilirubin exhibited a sensitivity, specificity, PPV, and NPV of 19.03%, 94.29%, 95.92%, and 14.16%, respectively. Similarly, for differentiating perforated appendicitis from non-perforated appendicitis, serum bilirubin levels showed sensitivity, specificity, PPV, and NPV of 70.59%, 80.97%, 43.37%, and 93.02%, respectively. Our findings are consistent with those of the study conducted by Kar et al. [18], except for the specificity for perforated appendicitis, which was higher in the present study.

Emmanuel et al. [19] reported that hyperbilirubinemia had a specificity of 88% for acute non-perforated appendicitis and 70% for gangrenous/perforated appendicitis, with significantly elevated levels in the latter (p < 0.01). In our study, hyperbilirubinemia exhibited a specificity of 94.29% for non-perforated versus normal appendices, and 80.97% for perforated versus non-perforated appendicitis. Mean bilirubin levels were significantly higher in patients with perforated appendicitis (p < 0.0001). Furthermore, in their study, hyperbilirubinemia demonstrated a PPV of 91% and CRP showed a specificity of 71% for acute non-perforated appendicitis. In our study, hyperbilirubinemia yielded a PPV of 95.92%, while CRP exhibited a specificity of 60% for non-perforated appendicitis.

Several studies have reported hyperbilirubinemia in appendicitis patients. Table 5 presents a comprehensive analysis of the sensitivity, specificity, PPV, and NPV of serum total bilirubin across multiple studies, including the present investigation.

**Table 5:** Comparison of serum total bilirubin’s sensitivity, specificity, positive predictive value, and negative predictive value for perforated appendicitis in multiple studies.

Authors	Study type	Number of subjects	Age (years, mean, range)	Histologically confirmed appendicitis (n)	Perforated appendix (n)	Sensitivity	Specificity	Positive predictive value	Negative predictive value
Present study	Retrospective study	333	31 years (3–87)	298	51	0.70	0.80	0.43	0.93
D’Souza et al. [20]	Prospective study	260	27.8 years (5–85)	110	20	0.70	0.82	0.47	0.93
Emmanuel et al. [19]	Retrospective study	472	27 years (5–82)	386	45	0.60	0.70	0.21	0.92
Sand et al. [17]	Retrospective study	538	36 years (6–91)	376	97	0.70	0.86	0.51	0.93
Bakshi et al. [11]	Prospective study	110	23.5 (5–62)	110	35	0.91	0.88	0.78	0.96
Kar et al. [18]	Prospective study	125	30.76 (4–70)	125	18	0.89	0.53	0.38	0.94
Estrada et al. [6]	Retrospective study	170	33 years (5–66)	157	41	0.56	0.69	0.39	0.82

In this study, patients with appendiceal perforation demonstrated significantly elevated mean bilirubin levels (p < 0.0001), corroborating the findings of Vaziri et al. [21] who reported similar findings.

Limitations

This study had several limitations owing to its retrospective design. Furthermore, the inclusion of only individuals who underwent appendicectomy may have influenced the specificity of the inflammatory markers. Additionally, being a single-center study with a small sample size further impacts its generalizability. Therefore, we recommend conducting a prospective study with preoperative bilirubin samples and a larger study population to overcome these limitations and strengthen our findings.

## Conclusions

In this study, we observed a significant difference in bilirubin levels between the patients diagnosed with appendicitis and those diagnosed with perforated appendicitis. Bilirubin can aid in diagnosis rather than being solely a confirmation tool. Elevated bilirubin levels in patients with appendiceal perforation suggest its potential as a reliable predictor, making it an important predictor of perforation. Therefore, we recommend including bilirubin levels in the evaluation of patients suspected of having acute appendicitis because of its simplicity, cost-effectiveness, and widespread availability. Bilirubin, in conjunction with clinical examinations and other laboratory tests, can enhance the evaluation of patients with suspected acute appendicitis.
